# Family Reading

**Published:** 1879-10

**Authors:** 


### FAMILY READING.

THE character, extent of culture and elevation of individuals and families is indexed by the newspapers, magazines and books which they habitually read; this reading confirms the character, makes it more pronounced, elevates or degrades. A Christian prefers religious books, and becomes a more decided Christian. A business man prefers a newspaper which most abounds in business information as to ships, merchandise, and stocks and bonds. The trifling mind busies itself in reading trashy novels and dishwater love stories. Every reflecting person will naturally select that kind of reading for himself and those under him which will cherish and cultivate the traits and dispositions which are most desired. Every family, then, ought to select its reading for the year with reflection and judgment; the father and mother are responsible for the household, the children and the servants under them.

Every religious family owes it to itself and its Church to take the religious paper which is nearest the authorized organ of the society to which it belongs; and a wrong is done to that Church if its paper is not patronized in preference to any other religious newspaper, however superior it is, for patronage will help to make it superior. There is no liberality, in the true sense of the term, for a man to take a paper of another denomination while he neglects the paper of his own Church, any more than it is true liberality and a wise policy to go and hear strange preachers, and attend other churches than your own, even if of the same denomination. He is the most liberal church member who oftenest goes to his own meeting and most sustains his own minister; these are the only church members who are really of any account in building up the Church anywhere, and making it an aggressive and a progressive organization.

If, on the other hand, a man wants his sons and daughters to grow up thoughtful, inquiring, intelligent, and well informed as to the duties of life, instead of bringing into the family a promiscuous assortment of fictitious reading, with illustrations of all that is violent and vile, he will sedulously and peremptorily exclude these from his centre table and patronize magazines of acknowledged merit, known to give substantial reading.

The farmer who does not take some agricultural publication will lose more in any one year than would pay the subscription price of a dozen, many times multiplied, even if he cultivates but a score or two acres of land.

There are a few papers which belong to the farm which give instruction, and receive information on a great variety of subjects. The names of a number of these will be found in previous numbers of the JOURNAL, with the places and prices of publication.

